# P-65. Clinical and microbiological characteristics of bacteremia in anti-interleukin-6 inhibitor recipients

**DOI:** 10.1093/ofid/ofaf695.294

**Published:** 2026-01-11

**Authors:** Koh Shinohara, Yusuke Tsuda, Yasuhiro Tsuchido, Masaki Yamamoto, Yasufumi Matsumara, Miki Nagao

**Affiliations:** Kyoto University Graduate School of Medicine, Kyoto, Kyoto, Japan; Kyoto University Graduate School of Medicine, Kyoto, Kyoto, Japan; Kyoto University Graduate School of Medicine, Kyoto, Kyoto, Japan; Kyoto University Graduate School of Medicine, Kyoto, Kyoto, Japan; Kyoto University Graduate School of Medicine, Kyoto, Kyoto, Japan; Kyoto University Graduate School of Medicine, Kyoto, Kyoto, Japan

## Abstract

**Background:**

Interleukin (IL)-6 plays a key role in the acute-phase response to infections. Use of anti-IL-6 inhibitors (anti-IL6) is associated with serious infections, but the characteristics of bacteremia in anti-IL6 recipients remain unclear.

**Figure 1.**
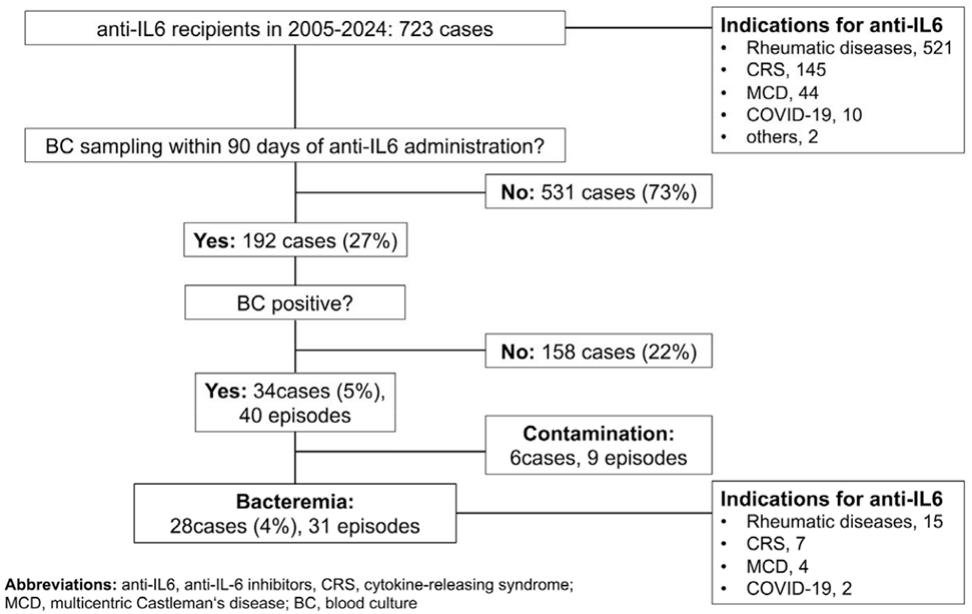
Flowchart of patients’ inclusion and exclusion

**Figure 2.**
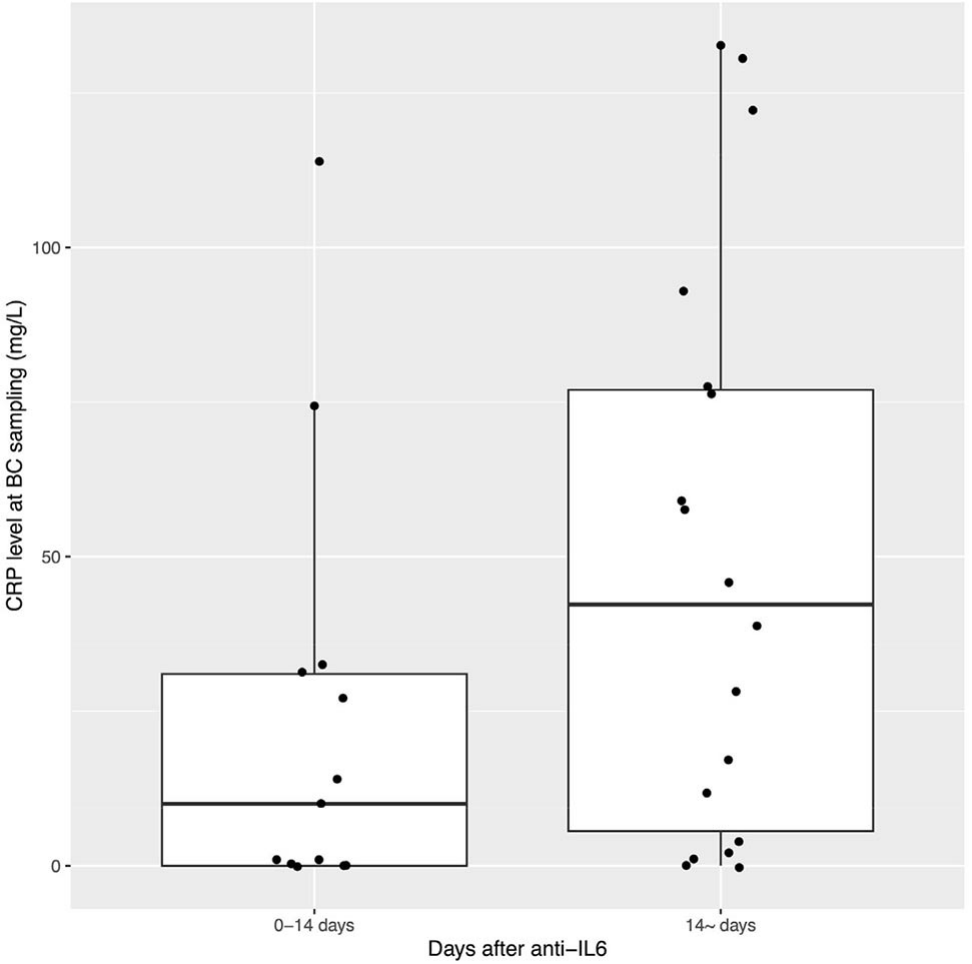
Boxplots and jitter plots of the CRP levels at the blood culture sampling according to the days after anti-IL6 administration

**Methods:**

We conducted a retrospective study in the Kyoto University Hospital, including all patients developing bacteremia within 90 days of anti-IL6 administration between 2005 and 2024. “Complicated bacteremia” was defined as meeting any of the following: (1) infective endocarditis; (2) multiple foci; (3) localized suppurative complications requiring surgical intervention; (4) bone and joint infections; or (5) persistent bacteremia.

**Results:**

A total of 31 episodes in 28 patients were included (Figure 1). Median age was 64 years; and 14 cases (50%) were male. Tocilizumab was given in 25 cases and sarilumab in 3 cases; indications were rheumatic diseases (15 cases), cytokine-releasing syndrome (7), Castleman’s disease (4) and COVID-19 (2). Additional immunosuppressive therapies were used in 89% of cases. In the 31 episodes, median time from last anti-IL6 administration to blood culture (BC) sampling was 19 days. Predominant foci were catheter-related bloodstream infections (n = 9) and intra-abdominal infections (9), followed by bone and joint infections (4). Sixteen episodes were caused by Gram-positives, thirteen by Gram-negatives, and two were mixed. Predominant pathogens were *Pseudomonas aeruginosa* and *Staphylococcus epidermidis* (5 episodes each), followed by *S. aureus* and *Escherichia coli* (4 episodes each). Eighteen episodes (58%) were nosocomial onset. Complicated bacteremia accounted for 13 episodes (42%), notably in 62% (8 of 13) of community-onset episodes and *S. aureus* bacteremia (3 of 4). C-reactive protein (CRP) levels at BC sampling were lower in episodes developing within 14 days of anti-IL6 compared to those after 15 days (23.5 mg/L vs 49.6 mg/L, p = 0.059; Figure 2). Antibiotic therapy over 14 days was administered in 20 episodes (65%). 30-day mortality was 11%.

**Conclusion:**

Over 40% of bacteremia episodes in anti-IL6 recipients were complicated, especially among community-onset cases. CRP levels were lower in episodes developing within 14 days of anti-IL6 administration.

**Disclosures:**

Yasufumi Matsumara, MD, PhD, Beckman Coulter: Research support for a collaborative project|Precision System Science: Research support for a collaborative project Miki Nagao, MD, PhD, Beckman Coulter: Research support for a collaborative project|Precision System Science: Research support for a collaborative project

